# Graft failure of IgA nephropathy in renal allografts following living donor transplantation: predictive factor analysis of 102 biopsies

**DOI:** 10.1186/s12882-019-1628-z

**Published:** 2019-12-03

**Authors:** Jin Zhang, Guo-dong Chen, Jiang Qiu, Guo-chang Liu, Li-zhong Chen, Kai Fu, Zi-xuan Wu

**Affiliations:** 1 Department of Urology, Guangzhou Women and Children’s Medical Center, Guangzhou, 510000 China; 2 Department of Organ Transplant, the First Affiliated Hospital of Sun Yat-sen University, Guangzhou, 510000 China

**Keywords:** IgA nephropathy, Graft failure, Living donor transplantation, Biopsy

## Abstract

**Background:**

To investigate predictive factors related to graft failure of IgA nephropathy(IgAN) in renal allografts following living donor transplantation.

**Methods:**

We identified a series of 102 biopsies diagnosed as IgAN in renal allografts following living donor transplantation from July 2004 to January 2017 at our center, and assess the predict value of the Lee’s classification and the 2009 Oxford classification in IgAN in renal allografts, clinical, ultrasonic and pathological characteristics at biopsy and the outcomes were retrospectively analyzed.

**Results:**

The 5-year graft cumulative survival rate after transplantation was 91.4%. The 4-year graft cumulative survival rate after biopsy diagnosis of IgAN in renal allografts was 59.6%. The mean time ± SD to disease was 4.7 ± 3.5 years. The color doppler ultrasound and blood flow imagine showed the echo enhancement, the reduced blood flow distribution, the reduced peak systolic velocity of main renal artery, and the increased resistance index of arcuate renal artery were valuable in evaluating the graft dysfunction. The Cox multivariate analysis revealed that the 24-h urinary protein level (HR 1.6 for 1-g increase, 95%CI 1.2–2.0), estimated glomerular filtration rate (eGFR) (HR 1.0 for 1-mL/min/1.73 m^2 decline, 95%CI 1.0–1.1), and mesangial C1q deposition (HR 3.0, 95%CI 1.2–7.4) at biopsy were independent predictive factors of graft failure of IgAN in renal allografts.

**Conclusions:**

IgAN in renal allografts occurred frequently within 5 years after transplantation. The risk of graft failure should be taken seriously in patients who exhibit heavy proteinuria and/or a declined eGFR as the initial symptoms; a high lesion grade (grade IV-V of Lee’s classification) and/or mesangial C1q deposition may also indicated a poor outcome.

## Background

The widespread use of effective immunosuppression has markedly reduced the risk of graft failure due to rejection in recent year s[1]. Conversely, de novo or recurrent renal disease has become a major cause of graft dysfunction, which was confirmed in our previous study,[2] and it is the second leading cause of death-censored graft failure and the third leading cause of uncensored graft failur e[3, 4].

IgA nephropathy (IgAN) is the most common recurrent nephropathy after transplantation, especially living donor transplantatio n[5, 6]. Some scholar s[5, 7] have suggested that IgAN recurrence is an independent risk factor for graft failure, but few studies have investigated the risk factors of IgAN in renal allografts that are predictive of disease progression and the outcomes compared to native IgA N[8, 9]. Therefore, the prognosis of IgAN in renal allografts remains controversia l[7, 10, 11]. The aim of this study was to investigate the predictive factors related to graft failure of IgAN in renal allografts following living donor transplantation.

## Methods

### Sample collection

In a total of 637 living donor transplantations in the First Affiliated Hospital of Sun Yat-sen University (Guangzhou, China) from July 2004 to January 2017, we identified a series of 102 patients with a diagnosis of IgAN in renal allografts proven by biopsy, including 93 indication biopsies and 9 protocol biopsies, repeated biopsies with a same diagnosis were excluded. The series consisted of 72 males and 30 females with a mean age ± SD of 35.5 ± 9.1 years. Sixty-seven patients have been proven primary IgAN by biopsy for native renal in this series.

A follow-up was performed in the First Affiliated Hospital of Sun Yat-sen University (Guangzhou, China) and Guangzhou Women and Children’s Medical Center (Guangzhou, China). Routine blood and urine examinations, liver and renal function tests, and therapeutic drug monitoring were conducted prior to biopsy for all patients. The standard maintenance therapy included a calcineurin inhibitor (CNI) (Tacrolimus was initiated at 0.05 mg/kg q12h or Cyclosporine A was initiated at 2–5 mg/kg q12h, the dosage adjusted according to the blood concentrations) in combination with mycophenolate mofetil (MMF) (Mycophenolate Sodium was initiated at 360 mg q12h) and corticosteroids. Oral prednisone was initiated at 30 mg (qd) and gradually reduced to 5–10 mg (qd) over a 4-week period. Table 1 lists other clinical characteristics and the therapeutic regimen after disease occurrence.

**Table 1:** Baseline characteristics of the recipients and donors

Variables	Graft survival (*n* = 76)	Graft failure (*n* = 26)	*p-*value
Recipient age (years)	34.1 ± 8.5	39.3 ± 9.7	0.05
Recipient sex, f/m (%)	18/58 (24/76)	9/17 (35/65)	0.27
Recipient race, East Asian (%)	76 (100)	26 (100)	–
Diabetes (%)	14 (18.4)	5 (19.2)	0.93
SBP (mmHg)	146.3 ± 17.6	149.5 ± 19.4	0.92
DBP (mmHg)	74.4 ± 9.5	75.9 ± 10.5	0.50
Acute rejection^a^ (%)	7 (9.2)	4 (15.4)	0.38
Delayed graft function (%)	4 (5.2)	2 (7.7)	0.65
Anti-HLA antibodies^b^ (%)	13 (17.1)	4 (15.4)	0.84
Class I (%)	5 (6.6)	3 (11.5)	0.42
Class II (%)	13 (17.1)	2 (7.7)	0.24
HLA mismatches (A, B, or DR)	2.1 ± 1.1	1.7 ± 1.0	0.77
Therapeutic regimen			
Intravenous methylprednisolone pulses^c^ (%)	21 (27.6)	7 (26.9)	0.94
Tonsillectomy (%)	10 (13.1)	6 (23.1)	0.23
ACEIs used (%)	59 (77.6)	23 (88.5)	0.23
Disease time^d^ (years)	4.0 ± 3.4	5.3 ± 3.7	0.62
Waiting time^e^ (months)	0.8 ± 1.3	0.4 ± 0.6	0.14
24-h urinary protein (g/24 h)	0.3 ± 0.2	0.4 ± 0.3	0.10
Urinary RBC count (u/ml)	1.5 ± 1.8	2.0 ± 2.5	0.22
Cholesterol (mmol/L)	5.3 ± 1.5	5.7 ± 2.5	0.36
LDL (mmol/L)	3.4 ± 1.1	3.8 ± 1.6	0.16
ALB (g/L)	42.0 ± 4.4	40.8 ± 4.4	0.24
eGFR^f^ (mL/min/1.73 m^2)^g^	75.2 ± 21.8	67.9 ± 20.7	0.14
Donor age (years)	50.4 ± 8.6	49.5 ± 8.4	0.77
Donor sex f/m (%)	53/23 (69/31)	14/12 (54/46)	0.14
Donor race, East Asian (%)	76 (100)	26 (100)	–

*SBP* Systolic blood pressure, *DBP* Diastolic blood pressure, *HLA* Human leukocyte antigen, *ACEIs* Angiotensin-converting enzyme inhibitors, *RBC* Red blood cell, *LDL* Low-density lipoprotein, *ALB* Albumin, *eGFR* Estimated glomerular filtration rate

^a^patients with a history of acute rejection after transplantation were included if they were cured when diagnosed as IgAN

^b^including donor-specific antibodies and non-donor specific antibodies

^c^three intravenous methylprednisolone pulses at the beginning of recurrence, but routine anti-rejection treatments after transplantation were excluded

^d^time from transplantation to the onset of initial symptoms

^e^time from the onset of the initial symptoms to biopsy

^f^baseline eGFR after transplantation

^g^the volume of urine filtrated by glomeruli every 1 min in a body surface area of 1.73 square metre

The Regional Ethics Committee of our center approved this study, and all patients signed informed consent forms.

### Biopsy and diagnosis of IgAN in renal allografts

The following indications for biopsy were combination of doctors’ experience and the 2009 KDIGO Clinical Practice Guideline for the Care of Kidney Transplant Recipient s[12] were used: 1) continuous anuria or oliguria (< 400 ml/24 h); 2) durative hematuria or proteinuria (positive in urine examinations for more than 1 month); 3) continuous increase in serum creatinine (sCr) (> 30% of the baseline) or a concentration above the normal level; 4) B-scan ultrasonography showing an abnormal blood flow peak systolic velocity (Vs) and resistance index (RI); and 5) panel-reactive antibody (PRA) level > 0% or the presence of donor-specific antibodies (DSAs).

Ninety-three patients with indications were suggested accept biopsy in 1 month, most of them (80.6%) underwent a timely biopsy, the mean ± SD waiting time for biopsy was 0.7 ± 1.2 months.

Nine patients with high risk factors for recurrence, such as a family history or primary IgAN diagnosed by native renal biopsy, underwent protocol biopsies at 6 months and at 1, 2, 5 and 10 years.

**Fig. 1:**
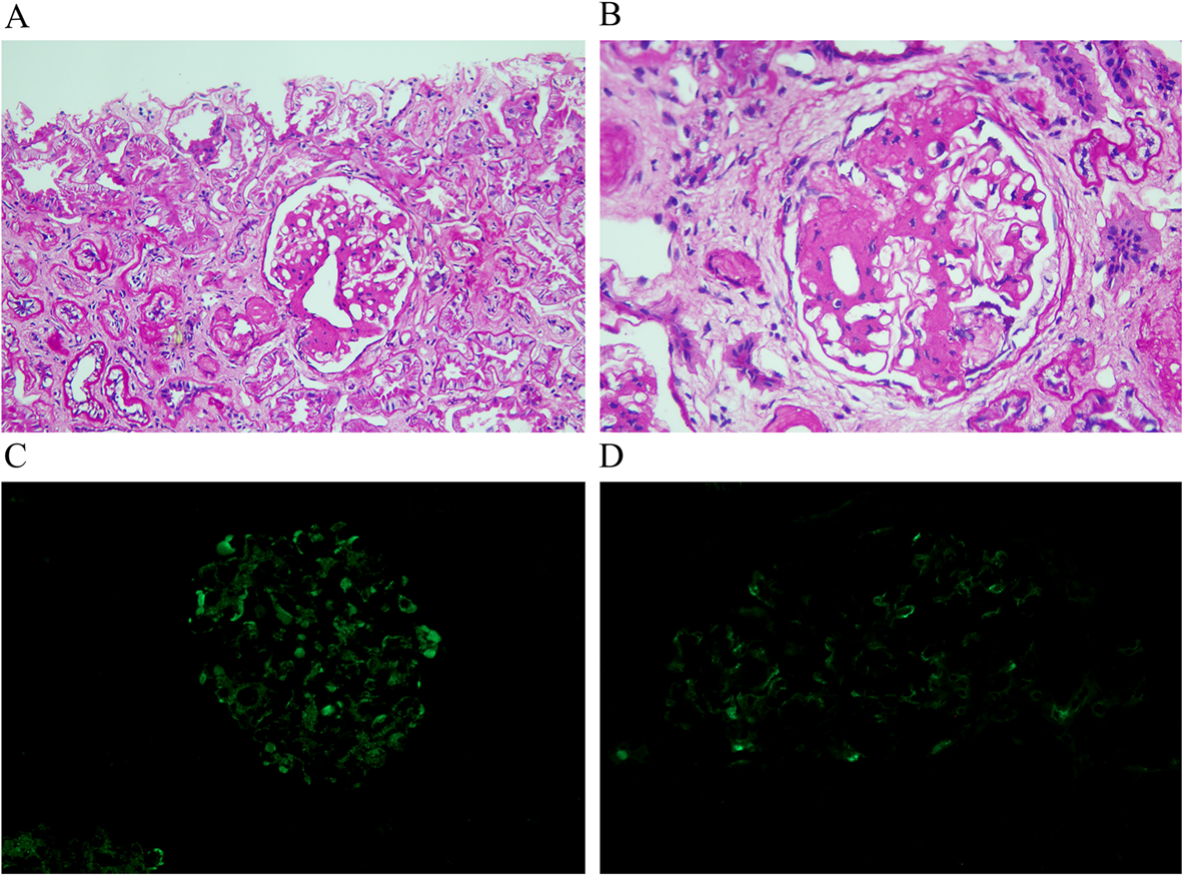
Pathological characters of IgAN in renal allografts (Lee’s IV, Oxford M1E1S1T1). **a** Glomerular mesangial proliferation and endocapillary hypercellularity, with part of the renal tubular atrophy. (PAS, ×200). **b** The basement membrane fragmented and absent, segment glomerular sclerosis and cellular/fibrocellular crescent formation. (PAS, ×400). **c** The positivity of mesangial IgA deposition. (Immunofluorescence staining). **d** The positivity of mesangial C1q deposition. (Immunofluorescence staining)

An ultrasonography-guided needle biopsy was performed using an 18-gauge needle (Bard). Each sample included at least 6 glomeruli visible by light microscopy. Immunofluorescence analyses were performed for all biopsies, and the IgA, IgG, IgM, C3, C1q and C4D levels were graded by two senior pathologists in an independent and blinded fashion. A diagnosis of IgAN in renal allografts based on IgA-positivity due to immunofluorescence staining deposition in the mesangial area (Fig. 1), which caused by lupus nephritis (LN) or other renal graft diseases was exclude d[13]. The classification were determined based on Lee’s and the 2009 Oxford classifications.

The ultrasonic data at the time of the biopsy diagnosis were recorded simultaneously, included cortical thickness, echo enhancement, peak systolic velocity (Vs) and RI in each renal artery.

### Statistical analysis

According to 2002 K/DOQI clinical practice guidelines for chronic kidney disease,[14] we defined the graft failure as the endpoint, which means that an estimated glomerular filtration rate (eGFR) less than 30 mL/min/1.73 m^2 (estimated by CKD-EPI Equation), without recovery for more than 3 months or returned to dialysis immediately.

We analyzed the time to disease recurrence, the 5-year graft cumulative survival rate after transplantation and the 4-year graft cumulative survival rate after biopsy diagnosis. Because the aim of this study was to investigate the predictive factors related to graft failure of IgAN in renal allografts rather than recurrence itself, we defined the initiation of the follow-up time from the day of biopsy diagnosis instead of the day of transplantation.

The urinary red blood cell (U-RBC) count, 24-h urinary protein level, serum albumin (ALB), eGFR, degree of histopathological injury, ultrasonic and other clinical characteristics at the time of the biopsy diagnosis were recorded. Based on Lee’s classifications, ultrasonic and blood flow index such as cortical thickness, echo enhancement, Vs and RI were compared in different degree of histopathological injury. Variables were compared using the Mann-Whitney U test or Chi-square test, and *p* values lower than 0.05 were considered significant. Correlation analyses were performed among abovementioned characteristics, Pearson’s and Spearman’s correlation coefficient were used for continuous and categorical variables respectively.

Univariate Cox proportional hazards models were used to investigate the significance of predictive factors related to graft failure, and significant factors were tested in the multivariate analysis. Kaplan-Meier estimates were used to create graft survival curves, and comparisons were performed using the log-rank test.

The data were analyzed using the IBM SPSS Statistics software version 22.0.

## Results

Ninety-three patients who were diagnosed with IgAN in renal allografts underwent a biopsy for initial symptoms. Proteinuria occurred in 72 patients (70.6%), hematuria occurred in 65 patients (63.7%), hypoproteinemia occurred in 18 patients (17.6%), and increased sCr (> 30% of the baseline) occurred in 28 patients (27.4%). Nine patients (8.8%) did not have abnormalities and were diagnosed using the protocol biopsy.

**Fig. 2:**
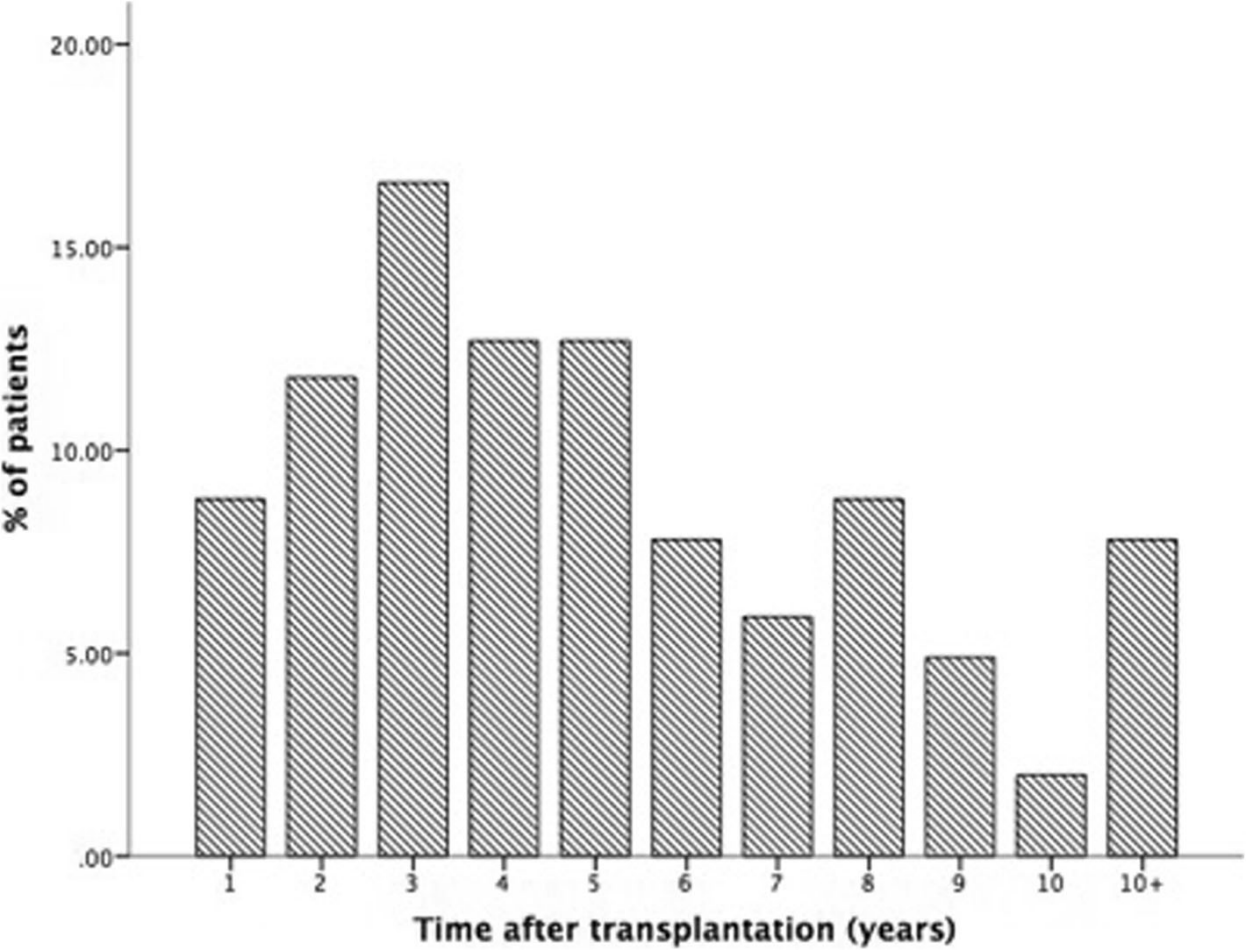
Duration from transplantation to IgAN in renal allografts

Graft failure occurred in 26 patients. The 5-year graft cumulative survival rate was 91.4% after transplantation (mean ± SD follow up, 7.6 ± 3.7 years), and the 4-year graft cumulative survival rate after diagnosis of IgAN by biopsy was 59.6% (the 5-year graft cumulative survival rate after diagnosis was not obtained, because the mean ± SD follow up was 3.4 ± 1.9 years). The mean time ± SD to occurrence was 4.7 ± 3.5 years. Sixty-four cases (62.7%) occurred within 5 years after transplantation, and 38 cases (37.2%) occurred after more than 5 years (Fig. 2).

The color doppler ultrasound and blood flow imagine showed the echo enhancement (*p* <  0.01), the reduced blood flow distribution (*p* = 0.02), the reduced Vs of main renal artery (MRA) (*p* = 0.04), and the increased RI of arcuate renal artery (ARA) (*p* = 0.04) were valuable in diagnosing and evaluating the graft dysfunction (Table 2). There was a positive correlation between Lee’s classifications and echo enhancement (Spearman’s correlation coefficient = 0.4, *p* <  0.01), the reduced blood flow distribution (Spearman’s correlation coefficient = 0.3, *p* <  0.01), the reduced Vs of MRA (Spearman’s correlation coefficient = 0.3, *p* = 0.03), and the increased RI of ARA (Spearman’s correlation coefficient = 0.3, *p* = 0.03).

**Table 2:** Color doppler ultrasound and blood flow index (based on Lee’s classification)

Variable	Lee’s classification (I-V) (*n* = 75^a^)					*p* value
	I (*n* = 19)	II (*n* = 13)	III (n = 26)	IV (*n* = 14)	V (*n* = 3)	
Cortical thickness1^ab^ (cm)	1.5 ± 0.3	1.5 ± 0.2	1.5 ± 0.2	1.6 ± 0.3	1.5 ± 0.2	0.40
Cortical thickness2^bc^ (cm)	1.7 ± 0.6	1.5 ± 0.3	1.5 ± 0.3	1.5 ± 0.3	1.3 ± 0.2	0.59
Change in cortical thickness^cd^ (cm)	−0.1 ± 0.6	0.1 ± 0.4	0 ± 0.2	0.1 ± 0.3	0.2 ± 0.1	0.23
Echo enhancement (%)	2 (10.5)	1 (7.7)	2 (7.7)	8 (57.1)	2 (66.7)	< 0.01
Blood flow distribution1 (I-V)	4.9 ± 0.3	4.8 ± 0.4	4.9 ± 0.3	4.9 ± 0.4	5.0 ± 0	0.96
Blood flow distribution2 (I-V)	4.8 ± 0.4	4.6 ± 0.6	4.7 ± 0.5	4.4 ± 0.7	3.7 ± 1.1	0.06
Change in blood flow distribution (I-V)	0.5 ± 0.2	0.2 ± 0.4	0.1 ± 0.4	0.5 ± 0.8	1.3 ± 1.1	0.02
MRA						
Vs1 (cm/s)	91.7 ± 20.0	109.4 ± 46.6	102.1 ± 26.8	100.1 ± 30.7	133.6 ± 45.7	0.31
Vs2 (cm/s)	104.6 ± 54.1	114.8 ± 48.3	117.2 ± 49.1	82.2 ± 32.1	95.4 ± 35.2	0.20
Vs1- Vs2 (cm/s)	−12.9 ± 61.1	−5.4 ± 26.7	−15.1 ± 51.4	17.9 ± 24.6	38.3 ± 10.5	0.04
RI1	0.6 ± 0.1	0.7 ± 0.1	0.7 ± 0.1	0.7 ± 0.1	0.6 ± 0	0.80
RI2	0.7 ± 0.1	0.7 ± 0.1	0.7 ± 0.1	0.7 ± 0.1	0.7 ± 0	0.36
RI1/RI2	1.0 ± 0.2	1.0 ± 0.1	1.0 ± 0.1	0.9 ± 0.1	0.9 ± 0.1	0.15
SRA						
Vs1 (cm/s)	54.2 ± 17.4	59.0 ± 18.1	55.3 ± 14.7	61.6 ± 19.0	81.3 ± 2.1	0.14
Vs2 (cm/s)	53.1 ± 15.1	60.6 ± 18.8	56.6 ± 13.0	52.4 ± 21.4	45.5 ± 3.5	0.38
Vs1- Vs2 (cm/s)	1.1 ± 20.8	−1.6 ± 12.6	−1.3 ± 17.7	9.1 ± 23.6	35.8 ± 5.3	0.05
RI1	0.6 ± 0.1	0.6 ± 0.1	0.6 ± 0.1	0.6 ± 0.1	0.6 ± 0.1	0.66
RI2	0.6 ± 0.1	0.6 ± 0	0.6 ± 0.1	0.7 ± 0.1	0.7 ± 0.1	0.15
RI1/RI2	1.0 ± 0.1	1.0 ± 0.1	1.0 ± 0.1	0.9 ± 0.1	0.9 ± 0.1	0.18
IRA						
Vs1 (cm/s)	29.4 ± 8.0	34.5 ± 9.1	31.2 ± 8.0	34.2 ± 9.1	37.4 ± 10.7	0.26
Vs2 (cm/s)	31.2 ± 10.3	34.5 ± 7.5	32.7 ± 8.5	28.6 ± 10.9	28.5 ± 10.5	0.42
Vs1- Vs2 (cm/s)	−1.8 ± 11.4	0 ± 9.3	−1.5 ± 10.2	5.6 ± 9.3	8.8 ± 8.3	0.10
RI1	0.6 ± 0.1	0.6 ± 0.1	0.6 ± 0.1	0.6 ± 0	0.6 ± 0	0.92
RI2	0.6 ± 0.1	0.6 ± 0.1	0.6 ± 0.1	0.6 ± 0.1	0.7 ± 0	0.11
RI1/RI2	1.0 ± 0.2	1.0 ± 0.1	1.0 ± 0.1	0.9 ± 0.1	0.9 ± 0	0.08
ARA						
Vs1 (cm/s)	20.8 ± 6.7	21.6 ± 4.4	19.7 ± 5.3	25.3 ± 7.2	27.4 ± 6.2	0.07
Vs2 (cm/s)	20.2 ± 8.0	22.1 ± 8.2	20.9 ± 9.5	18.9 ± 8.0	19.6 ± 4.5	0.79
Vs1- Vs2 (cm/s)	0.6 ± 7.3	−0.5 ± 9.3	−1.2 ± 13.0	6.4 ± 9.1	7.8 ± 1.8	0.07
RI1	0.6 ± 0	0.6 ± 0	0.6 ± 0.1	0.6 ± 0	0.6 ± 0	0.85
RI2	0.6 ± 0.1	0.6 ± 0.1	0.6 ± 0.1	0.6 ± 0.1	0.7 ± 0.1	0.04
RI1/RI2	1.0 ± 0.1	1.0 ± 0.1	1.1 ± 0.2	1.0 ± 0.1	0.9 ± 0	0.04

^a^in total series, 27 cases without complete ultrasonic data

^b^the baseline level at the time when patients had the lowest sCr level after transplantation, the same definition for following index

^c^the level at the time of biospy, the same definition for following index

^d^the change from baseline level to the level at the time of biospy, the same definition for following index

The univariate analysis revealed that age (*p* = 0.05), time to disease occurrence (*p* = 0.05), the 24-h urinary protein level (*p* <  0.01), eGFR (*p* <  0.01), hypoproteinemia (ALB < 35.0 g/L) (*p* <  0.01), Lee’s classification (*p* = 0.02), mesangial C1q deposition (*p* <  0.01) at the time of biopsy were predictive of graft failure. We found significant correlations between the abovementioned characteristics. For instance, the time to disease occurrence was directly correlated with age (Pearson’s correlation coefficient = 0.4, *p* <  0.01), Lee’s classification (Spearman’s correlation coefficient = 0.3, *p* <  0.01), and mesangial C1q deposition (Spearman’s correlation coefficient = 0.2, *p* = 0.02). The 24-h urinary protein level was directly correlated with hypoproteinemia (Spearman’s correlation coefficient = 0.4, *p* <  0.01). The multivariate analysis demonstrated that the 24-h urinary protein level, eGFR and mesangial C1q deposition at the time of the biopsy were independent predictive factors related to graft failure. The hazard ratios (HRs) were 1.6 for 1-g increased 24-h urinary protein (95%CI 1.2–2.0), 1.0 for 1-mL/min/1.73 m^2 declined eGFR (95%CI 1.0–1.1), 3.0 for mesangial C1q deposition (95%CI 1.2–7.4) (Table 3).

**Table 3:** Predictive factors related to graft failure of IgAN in renal allografts

Predictive factors	Univariate		Multivariate	
	HR (95% CI)	*p* value	HR (95% CI)	*p* value
Age (years)	1.0 (1.0–1.1)	0.03	–	0.38
Disease time (years)	1.1 (1.0–1.3)	0.03	–	0.38
Waiting time^a^ (months)	0.7 (0.4–1.2)	0.20	–	–
SBP (mmHg)	1.0 (0.9–1.0)	0.90	–	–
DBP (mmHg)	1.0 (1.0–1.1)	0.55	–	–
24-h urinary protein (g/24 h)	1.6 (1.3–2.0)	< 0.01	1.6 (1.2–2.0)	< 0.01
Hematuria (− to +++)				
-	1.0	0.80	–	–
+	0.6 (0.2–1.7)	0.38	–	–
++	0.7 (0.2–1.9)	0.49	–	–
+++	0.7 (0.1–3.1)	0.63	–	–
Urinary RBC count (u/ml)	1.0 (0.9–1.0)	0.68	–	–
Cholesterol (mmol/L)	1.0 (0.8–1.2)	0.79	–	–
LDL (mmol/L)	1.1 (0.8–1.4)	0.57	–	–
ALB (g/L)	0.9 (0.8–0.9)	< 0.01	–	0.50
eGFR^b^ (mL/min/1.73 m^2)^c^	1.0 (1.0–1.1)	< 0.01	1.0 (1.0–1.1)	< 0.01
Therapeutic regimen				
MPP(%)	2.2 (0.9–5.5)	0.09	–	–
Tonsillectomy (%)	0.8 (0.3–2.0)	0.69	–	–
ACEIs used (%)	1.9 (0.6–6.2)	0.31	–	–
Lee’s classification (I-V)				
I	1.0	0.02	–	0.52
II	1.2 (0.3–4.9)	0.83	–	0.44
III	1.4 (0.4–4.5)	0.54	–	0.91
IV	4.2 (1.4–13.1)	0.01	–	0.97
V	5.6 (1.3–23.6)	0.02	–	0.09
Oxford classification (MEST)				
M0	1.0	–	–	–
M1	1.5 (0.2–11.1)	0.69	–	–
E0	1.0	–	–	–
E1	2.1 (0.9–4.5)	0.63	–	–
S0	1.0	–	–	–
S1	1.1 (0.4–2.8)	0.88	–	–
T0	1.0	–	–	–
T1–2	3.0 (0.7–12.7)	0.14	–	–
Immunofluorescence staining				
IgA +	–	–	–	–
IgG +	1.4 (0.5–4.0)	0.49	–	–
IgM +	2.5 (0.9–6.7)	0.26	–	–
C3 +	0.1 (0.4–2.2)	0.85	–	–
C1q +	4.0 (1.7–9.3)	< 0.01	3.0 (1.2–7.4)	0.02
C4d +	0.4 (0.1–2.8)	0.34	–	–
Comorbidity				
Acute rejection	1.5 (0.5–4.4)	0.45	–	–
Chronic rejection	6.7 (0.8–53.1)	0.07	–	–
CNI toxicity	2.3 (0.5–9.9)	0.25	–	–
BKVAN	–	0.69	–	–

*HR* Hazard ratio, *CI* Confidence interval, *SBP* Systolic blood pressure, *DBP* Diastolic blood pressure, *RBC* Red blood cell, *LDL* Low-density lipoprotein, *ALB* lbumin; *eGFR* Estimated glomerular filtration rate, *MPP* Methylprednisolone pulse, *ACEIs* Angiotensin-converting enzyme inhibitors, *CNI* Calcineurin inhibitor, *BKVAN* BK virus-associated nephropathy

^a^the time from onset of initial symptoms to biopsy

^b^the eGFR at the time of the biopsy

^c^the volume of urine filtrated by glomeruli every 1 min in a body surface area of 1.73 square metre

**Fig. 3:**
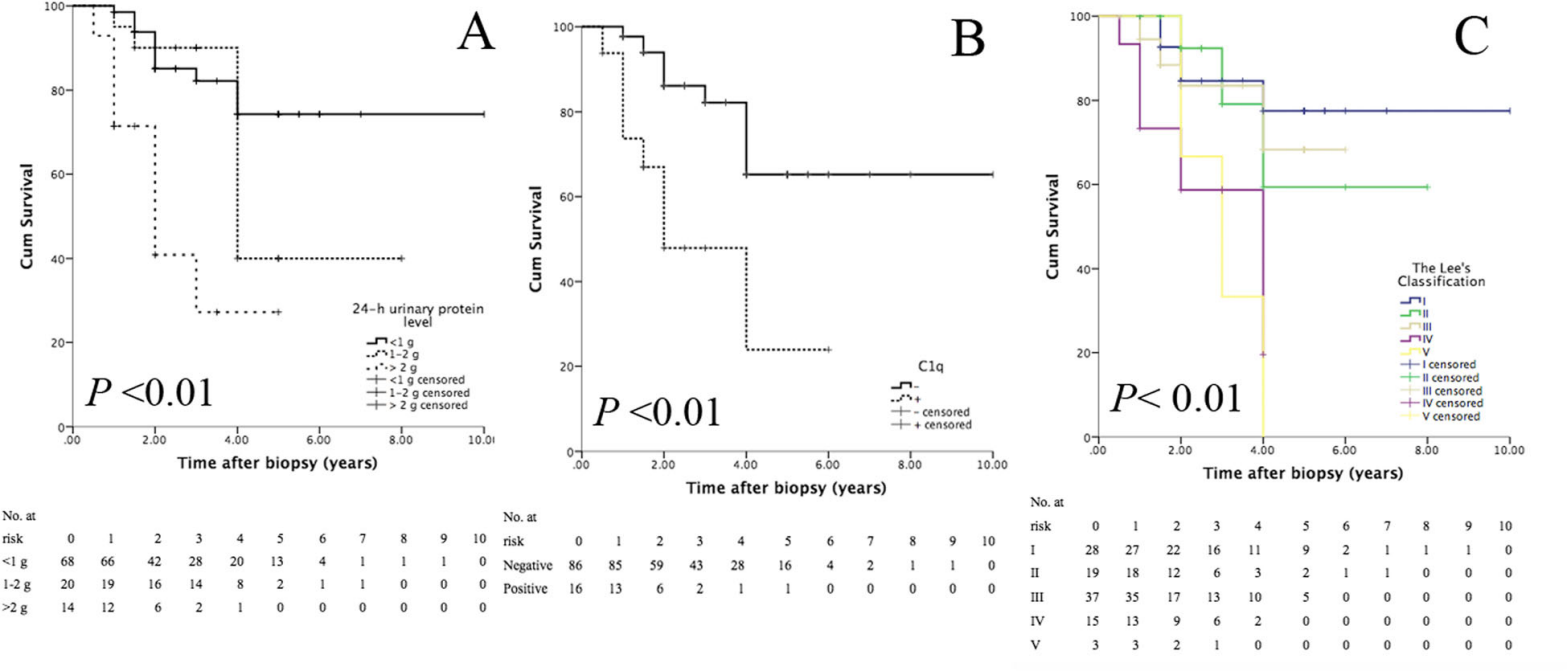
Kaplan-Meier curves of the 24-h urinary protein level (**a**), mesangial C1q deposition (**b**) and Lee’s classification (**c**)

Although some characteristics were not independent predictive factors of graft failure in our study, the log-rank test revealed a significant difference between the Kaplan-Meier curves of Lee’s classification (*p* <  0.01) (Table 4). Figure 3 shows the significant differences in the 24-h urinary protein level (*p* <  0.01) and other factors.

**Table 4:** The 4-year graft cumulative survival rate after biopsy diagnosis of different predictive factors

Predictive factor	Graft cumulative survival rate after biopsy diagnosis				
	1-year	2-year	3-year	4-year	Log-rank *p*
24-h urinary protein (g/24 h)					
< 1 (*n* = 68)	98.5%	85.0%	82.1%	74.3%	
1–2 (*n* = 20)	95.0%	90.0%	90.0%	40.0%	
> 2 (n = 14)	71.4%	40.8%	27.2%	27.2%	< 0.01
C1q deposition					
- (*n* = 86)	97.7%	86.1%	82.2%	65.2%	
+ (*n* = 16)	73.7%	47.8%	47.8%	23.9%	< 0.01
Lee’s classification (I-V)					
I (*n* = 28)	100%	84.5%	84.5%	77.5%	
II (n = 19)	100%	92.3%	79.1%	59.3%	
III (*n* = 37)	94.4%	83.4%	83.4%	68.3%	
IV (*n* = 15)	73.3%	58.7%	58.7%	19.6%	
V (n = 3)	100%	66.7%	33.3%	0	< 0.01

## Discussion

Due to differences in diagnostic criteria, discrepancies have been reported in recurrence rates, with a range from 9 to 61 %[5]. Family history of IgAN is a known factor of its recurrence,[15] combined with a review of the Australia-New Zealand registry,[6] IgAN patients accepted low HLA-mismatched living donor transplantation with higher risk of recurrence, thus the family history in cases who accepted related donors should be given more attention.

More and more recent studies confirmed that IgAN in renal allografts had impact on long-term graft survival,[9, 10, 16] which were inconsistent with early research suggested that it exhibited slow progression and benign outcomes,[11] especially in patients with asymptomatic hematuria and/or proteinuria. Floege et al .[17] reported that ∼5% of patients lost their grafts due to occurrence at 5 years after transplantation, and our study demonstrated a similar result. However, the 4-year graft cumulative survival rate was only 59.6% after the IgAN diagnosis, which showed a worse outcome in our study.

We found that IgAN occurred frequently within 5 years postoperatively and then declined sharply in subsequent years, which was consistent with a previous repor t[18]. Few patients diagnosed with IgAN in renal allografts showed a remarkable increased sCr level when accepted biopsy,[2] as reported in previous study on IgAN in renal allografts, it had little impact on graft function in the early years after transplantation,[19] but Ortiz et al .[20] found that the histopathological injury was not accompanied by abnormalities in the urinalysis in half of early recurrent IgAN patients. Therefore, the disease progression could be concealed, a protocol biopsy was recommended to prevent missed diagnoses, especially in clinically silent patients.

An increased sCr level or declined eGFR as the initial symptoms was generally associated with a high risk of graft failure. This result was confirmed in a previous study on native IgA N[21]. Another independent predictive factor related to renal failure in native and graft IgAN was the 24-h urinary protein level, which was confirmed in our and other recent studie s[22, 23]. This risk was obviously increased when proceeding to hypoproteinemia. Hematuria is a typical clinical symptom of IgAN, and one prognostic factor study in Chinese patients concluded that hematuria was a useful marker for patients who were at high risk for disease progression of native IgA N[24]. However, no correlation was found between hematuria and graft failure in our study, possibly for the following reasons: 1) IgAN in renal allografts is not the only cause of hematuria following transplantation, which is different than native IgAN, and drug toxicity damage, infection, use of a Double-J catheter and other factors may induce hematuria in these patients; 2) some patients with initial asymptomatic hematuria did not accept a biopsy in a timely manner; and 3) only 26 cases of graft failure were observed in this series. Thus, more samples and a longer-term follow-up are needed to test the predictive value of hematuria.

The color doppler ultrasound is helpful to evaluate the progress of renal graft diseases, the change in cortical thickness, echo enhancement and the reduced blood flow distribution are typical features of graft dysfunction, like echo enhancement denotes glomerulosclerosis and interstitial fibrosis, which showed in our results. The results indicate that patients with severe histopathological injury (grade IV-V of Lee’s classification) may show lower Vs and higher RI in blood flow than mild or moderate (grade I-III), it coincide partly with early research,[25] which is concluded that it can be diagnosed as renal disease when RI > 0.65 in interlobar renal artery (IRA), but we found that the RI fluctuated between 0.6 and 0.7 in most cases of IgAN in renal allografts, even if they had a good graft function, moreover, the blood flow in MRA and ARA had more significant changes than it in IRA. These differences may be concerned with the baseline level influenced by donor factors, in addition, handlers’ skills and experience are important influence factors.

Lee’s classification and the Oxford classification are useful prognostic indicators of disease progression and clinical outcomes of native IgAN, but their utility in IgAN in after transplantation remains unknown. Lee’s classification includes criteria for histopathological grading, such as glomerular sclerosis, crescent formation, interstitial inflammation and tubular atrophy, which were established based on and considered suitable for East Asian patients. Patients with mild or moderate (grade I-III) lesions have been reported to have a benign outcome, whereas patients with grade IV or V lesions develop end-stage renal failure,[26] which is completely consistent with our results. Thus, Lee’s classification exhibits the same utility for prediction of the outcome of IgAN in renal allografts. The 2009 Oxford classification includes the following histological components: mesangial (M) and endocapillary (E) hypercellularity, segmental sclerosis (S) and interstitial fibrosis/tubular atrophy (T). This classification provided a clear definition and accurate criteria for the various pathological lesions of IgAN. It is widely used, and its utility has been validated in many studie s[27, 28]. However, the advantages of this classification system for the clinical prediction of IgAN in renal allografts was not observed in our study. We considered that immunosuppressive therapy and donor factors might influence the pathological lesions represented by the four parameters in the Oxford classification. One study confirmed that immunosuppressive therapy reduced the predictive value of the pathological parameters in recurrent IgA N[28] compared with native IgAN. A simple “present” or “absent” for these parameters, such as M and E, cannot precisely reflect the degree of histopathological graft injury. Therefore, these parameters exhibit lower specificity in patients who accept long-term immunosuppressive therapy. The significance of crescent formation was not listed as a prognostic parameter in the 2009 Oxford classification, but in many follow-up validation studie s[29, 30] this parameter was considered prognostic, as a result, the Oxford classification was updated to MEST-C (crescent) in 201 7[31]. This study is the first report to assess the predictive value of these two classifications for IgAN in renal allografts, and the advantages and disadvantages of these classifications should be further assessed. We support the use of a combination of the pathological parameters with clinical features at the time of biopsy to provide earlier risk prediction in IgAN.

Sixteen cases with mesangial C1q deposition in this series. Traditionally, the presence of C1q staining is viewed as a typical pathological lesion of LN rather than IgA N[13]. However, a more recent stud y[32, 33] confirmed that this parameter played a role and occurred in 0 to 45% of patients with IgAN. Lee et al .[32] concluded that mesangial C1q deposition in the glomerulus was associated with a poor renal outcome and severe pathological features in native IgAN. We demonstrated that this parameter exhibited a higher predictive value in IgAN after transplantation. The absence of C1q deposition is a positive predictive sign as a response to steroid pulse therapy and relief of proteinuri a[33]. Therefore, steroid-resistance nephrotic syndrome and a poorer outcome should be taken seriously when C1q staining presents during long-term immunosuppression.

Maintenance therapy with medium/low prednisone is used to relieve IgAN progression in combination with angiotensin-converting enzyme inhibitors (ACEIs) to reduce proteinuria. Early steroid withdrawal is a safe intervention in living donor transplantation,[34, 35] but steroid withdrawal should be handled with caution when choosing maintenance immunosuppressive treatments for patients with a high risk of recurrence. Based on our experience of treatment of native IgAN and related references,[36, 37] a maintenance therapy of oral prednisone at a dose of 0.6 mg/kg (qod) for Chinese patients after a IgAN in renal allografts diagnosis may be worthwhile. One recent stud y[38] suggested that other immunosuppressive therapy did not improve outcomes in patients with native IgAN and exhibited a more limited effect in IgAN in renal allografts, which occurred under existing immunosuppression after transplantation. Moreover, patients with IgAN in renal allografts exhibited favorable outcomes after tonsillectomy,[39, 40] but no reliable treatment strategy was proven to cure it.

Our study analyzed the 5-year graft cumulative survival rates after transplantation, but longer-term follow-up from biopsy is needed to observe more outcome events. In most cases, the primary diseases of the recipients were unknown, only 67 cases have been proven primary IgAN by biopsy for native renal in this series, the differences in clinical and pathological characteristics between de novo and recurrent IgAN couldn’t be found in this study. Although major confounding variables were adjusted and our results were consistent with other studies in the literature, bias was unavoidable in the univariate Cox proportional hazards models due to the influence of the lack of information for the intraoperative and donor factors.

## Conclusion

IgAN is the most common de novo or recurrent nephropathy, especially in living donor transplantation, and occurs frequently within 5 years after transplantation. The color doppler ultrasound and blood flow imagine were valuable in diagnosing and evaluating the graft dysfunction. The risk of graft failure should be taken seriously in patients who exhibit heavy proteinuria (24-h urinary protein level > 2 g) and/or a declined eGFR as the initial symptoms, especially with hypoproteinemia, a high lesion grade (grade IV-V of Lee’s classification) and/or mesangial C1q deposition.

## Data Availability

All data and materials were collected from clinical and pathological database of the First Affiliated Hospital of Sun Yat-sen University and Guangzhou Women and Children’s Medical Center. The datasets used and/or analyzed during the current study are available from the author Dr. Jin Zhang (E-mail: m18205188630@163.com) on reasonable request.

## Abbreviations

ACEIs: Angiotensin-converting enzyme inhibitors
ALB: Albumin
ARA: Arcuate renal artery
BKVAN: BK virus-associated nephropathy
CI: Confidence interval
CR: Chronic rejection
CNI: Calcineurin inhibitor
DSAs: Donor-specific antibodies
HRs: Hazard ratios
IgAN: IgA nephropathy
IRA: Interlobar renal artery
KDIGO: Kidney Disease: Improving Global Outcomes
LDL: Low-density lipoprotein
LN: Lupus nephritis
MMF: Mycophenolate mofetil
MRA: Main renal artery
PRA: Panel-reactive antibody
RI: Resistance index
sCr: Serum creatinine
SRA: Segmental renal atery
U-RBC: Red blood cell
Vs: Peak systolic velocity
